# Administration of a VEGFR‑2-specific MRI contrast agent to assess orthodontic tooth movement: A pilot study

**DOI:** 10.1007/s00056-021-00326-x

**Published:** 2021-07-16

**Authors:** Agnes Schröder, Lisa Seyler, Elisabeth Hofmann, Lina Gölz, Jonathan Jantsch, Peter Proff, Tobias Bäuerle, Christian Kirschneck

**Affiliations:** 1 Department of Orthodontics, University Hospital Regensburg, 93053 Regensburg, Germany; 2 PIPE (Preclinical Imaging Platform Erlangen) and Department of Radiology, University of Erlangen-Nuremberg, 91054 Erlangen, Germany; 3 Private Orthodontic Practise, 44649 Herne, Germany; 4 Department of Orthodontics, University of Erlangen-Nuremberg, 91054 Erlangen, Germany; 5 Institute of Clinical Microbiology and Hygiene, University Hospital Regensburg, 93053 Regensburg, Germany

**Keywords:** Periodontal ligament, Vascular endothelial growth factor receptor‑2, Molecular magnetic resonance imaging, Hypoxia, Animal models, Parodontalligament, Vaskulärer endothelialer Wachstumsfaktor-Rezeptor‑2 (VEGFR-2), Molekulare Magnetresonanztomographie, Hypoxie, Tiermodelle

## Abstract

**Purpose:**

It is thought that orthodontic forces initially reduce periodontal blood flow during orthodontic tooth movement (OTM) via tissue compression with cells responding to concomitant oxygen deprivation with expression of vascular endothelial growth factor (VEGF) triggering angiogenesis via binding to its receptor VEGFR‑2. To test this hypothesis, we performed a pilot study to establish a protocol for molecular magnetic resonance imaging (MRI) of rat jaws administering a VEGFR-2-specific contrast agent.

**Methods:**

Mesial OTM of a first upper left rat molar was initiated in one male Fischer 344 rat 4 days prior to MRI by insertion of an elastic band between the first and second upper molars with the contralateral side left untreated (internal control). T1-weighted MRI sequences including dynamic contrast-enhanced MRI (DCE-MRI) were recorded before and after administration of a molecular VEGFR‑2 MRI marker with a 7 T MRI dedicated for small animal use.

**Results:**

After injection of anti-VEGFR2-albumin-gadolinium-DTPA, volume enhancement on T1-weighted images was increased at the OTM side distally of the moved first upper molar (M1) compared to the control side, whereas the T1 relaxation time was reduced on the OTM side. DCE-MRI resulted in an increased area under the curve (AUC), whereas time-to-peak (TTP) and washout rate were reduced during OTM distally of the moved M1 compared to the contralateral side.

**Conclusions:**

OTM resulted in uptake of the VEGFR-2-specific MRI contrast agent in tension areas of the periodontal ligament. The imaging protocol presented here is useful for the assessment of VEGFR‑2 expression in tension areas of the periodontal ligament in vivo.

## Abstract (de)

**Zielsetzung:**

Es wird angenommen, dass kieferorthopädische Kräfte initial den parodontalen Blutfluss während der kieferorthopädischen Zahnbewegung (OTM) durch Gewebekompression reduzieren, wobei die beteiligten Zellen auf einen begleitenden Sauerstoffmangel mit einer Expression des vaskulären endothelialen Wachstumsfaktors (VEGF) reagieren, der über die Bindung an seinen Rezeptor VEGFR‑2 die Angiogenese induziert. Um diese Hypothese zu untersuchen, führten wir eine Pilotstudie durch, um ein Protokoll für die molekulare Magnetresonanztomographie (MRT) von Rattenkiefern nach Verabreichung eines VEGFR-2-spezifischen Kontrastmittels zu etablieren.

**Material und Methoden:**

Bei einer männlichen Fischer-344-Ratte wurde der erste obere Molar (M1) auf der linken Kieferseite 4 Tage vor der MRT durch Einbringen eines elastischen Bandes zwischen dem ersten und zweiten oberen Molaren mesialisiert, wobei die kontralaterale Seite unbehandelt blieb (interne Kontrolle). T1-gewichtete MRT-Sequenzen einschließlich dynamischer kontrastmittelunterstützter MRT (DCE-MRT) wurden vor und nach der Verabreichung eines molekularen VEGFR-2-MRT-Markers mit einem 7‑Tesla-MRT für Kleintiere aufgezeichnet.

**Ergebnisse:**

Nach Injektion von Anti-VEGFR2-Albumin-Gadolinium-DTPA war die Volumenanhebung auf T1-gewichteten Bildern auf der OTM-Seite distal des bewegten ersten oberen Molaren (M1) im Vergleich zur Kontrollseite erhöht, während die T1-Relaxationszeit auf der OTM-Seite verkürzt war. Die DCE-MRT führte zu einer erhöhten Fläche unter der Kurve (AUC), während die TTP-Zeit bis zum Spitzenwert und die Auswaschungsrate während der OTM distal des bewegten M1 im Vergleich zur kontralateralen Seite reduziert waren.

**Schlussfolgerungen:**

Die kieferorthopädische Zahnbewegung führte zur Aufnahme des VEGFR-2-spezifischen MRT-Kontrastmittels in Zugzonen des Parodontalligaments. Das hier vorgestellte Bildgebungsprotokoll hat sich für die In-vivo-Beurteilung der VEGFR-2-Expression in Zugzonen des Parodonts als nützlich erwiesen.

## Introduction

When orthodontic forces are applied, compression and tension areas develop in the periodontal ligament. This initiates a complex molecular cascade involving multiple molecular and inflammatory mediators [15, 22, 30], released by mechanically stressed periodontal ligament fibroblasts [4], osteoblasts and cells of the immune system such as macrophages and T cells [29]. Stimulation of mechanosensitive ion channels and receptors in the cell membrane occurs [3]. Cells in the periodontal ligament appear to respond to mechanical stimuli by upregulating cellular mediators such as cyclic AMP, which catalyzes the phosphorylation of mediator proteins [5, 15]. Depending on these, either cell proliferation or cell differentiation may be stimulated within the cell nucleus. This is controlled by the phosphorylation of transcription factors, such as c‑Jun and c‑Fos [3]. If inflammatory processes are involved, cyclooxygenase 2 (COX-2) can produce prostaglandin E2 (PGE2), which plays an important role in bone resorption [21]. Inflammatory processes continue to stimulate mononuclear phagocytic cells, such as macrophages. These secrete proinflammatory cytokines which stimulate the secretion of prostaglandins [2]. During the application of orthodontic forces, these cytokines appear to stimulate the secretion of the receptor activator of nuclear factor kappa B ligand (RANKL) by osteoblasts and periodontal ligament cells in periodontal tissue [20, 31] and osteocytes in the alveolar bone [19, 23]. Recent studies provide evidence that osteocytes are critically involved in OTM via expression of RANKL. RANKL is essential for the differentiation of osteoclast precursor cells into active multinucleated osteoclasts, which are responsible for bone resorption.

However, orthodontic forces not only have cellular effects, but also affect the circulation within the periodontal ligament. Compression of blood vessels, especially in pressure areas during orthodontic force application, is assumed to effect reduced perfusion and thus hypoxia, i.e., a reduced supply of oxygen of the periodontal tissue below physiological levels [8, 18]. Depending on the oxygen gradient, either the proliferation of different cells is stimulated or, in extreme cases, apoptosis [18]. Hypoxia has a direct influence on the energy balance within the cell via a decrease in glycolytic activity and ATP production. The cell responds to oxygen deprivation by expression of various cellular mediators, such as hypoxia-induced factor‑1 (HIF-1), which stimulates angiogenesis and cell proliferation [8].

In addition, hypoxia has a special significance for transcriptionally regulated VEGF (vascular endothelial growth factor) expression. Thus, hypoxia via an increase in the HIF-1α level is the relevant inducer for the gene expression of VEGF under various pathophysiological conditions [14, 16, 24]. VEGF is an important signalling molecule that is effective in both vasculogenesis and angiogenesis. As the name implies, this factor mainly stimulates vascular endothelium, but it also stimulates the migration of monocytes and macrophages. Seven different forms of VEGF are known (A‑F and PIGF) [32]. All members of the VEGF family effect a cellular response by binding to a tyrosine kinase, the VEGF receptor (VEGFR), thus, relaying the extracellular signal to the cell interior. The stimulation of VEGF by binding to one of its receptors, VEGFR‑2, triggers the proliferation and migration of endothelial cells, as well as the increase of vascular permeability by activation of various signalling pathways (phosphatidylinositol 3’kinase [PI3K]/Akt and Ras/mitogen activated protein kinase [MAPK]), thus, effecting increased angiogenesis and in turn improving perfusion and oxygen supply within the periodontal ligament [7].

Despite the supposed effects of orthodontic force application on local microcirculation within the periodontal ligament and concomitant hypoxia-mediated angiogenesis during orthodontic tooth movement (OTM), it is still unclear to what extent local perfusion is actually altered during OTM and how this impacts on associated VEGF-induced angiogenesis within the periodontal ligament. For this reason, we performed a pilot study in the animal model rat using magnetic resonance imaging (MRI) to establish an imaging protocol for administration and detection of a VEGF receptor 2 (VEGFR-2)-specific MRI contrast agent within the periodontal ligament of an orthodontically moved upper rat molar.

## Materials and methods

### General information regarding the animal experiment

One male Fischer 344 rat (Rattus norvegicus Berkenhout, Charles River Laboratories, Sulzfeld, Germany) was included in this pilot study. The rat was 7 weeks old at the beginning of the experiment, which was carried out with the approval of the responsible authorities (Government of Lower Franconia, AZ: 55.2.2532-2-510) and in compliance with the German Animal Protection Act. In order to avoid unnecessary animal suffering, corresponding termination criteria were predefined and animal condition as well as gross body weight monitored daily. The animal was kept in a conventional S1 animal laboratory at the University of Erlangen-Nuremberg (Preclinical Experimental Animal Centre PETZ) and had ad libitum access to tap water and to a standard rat diet (V1535, ssniff) with feed pellets. After 4 days of OTM and after the respective mMRI (molecular magnetic resonance imaging) measurements, the rat was euthanized by an i.p. injection of 200 mg Narcoren per kg gross body weight (Merial GmbH, Hallbergmoos, Germany) according to legal guidelines.

### Induction of orthodontic tooth movement

After 7 days of acclimatization, the rat was sedated by intraperitoneal (i.p.) injection of 6 mg xylazine and 90 mg ketamine per kg gross body weight [11] and an orthodontic elastic band (774-200-01, Dentaurum, Ispringen, Germany) was inserted into the approximal space between the first and second upper left molar of the rat according to the method described by Waldo and Rothblatt ([27]; Fig. 2a). The contralateral jaw side was left untreated and served as nonforce internal control [11]. The renewed expansion of the compressed elastic band caused a reciprocal orthodontic force and divergence of the first and second upper left molars and thus an anterior experimental tooth movement of the first upper left molar for 4 days. After insertion, the oral cavity was disinfected with a cotton pellet soaked in chlorhexidine.

### Magnetic resonance imaging without/with an VEGFR-2 marker

**Fig. 1:**
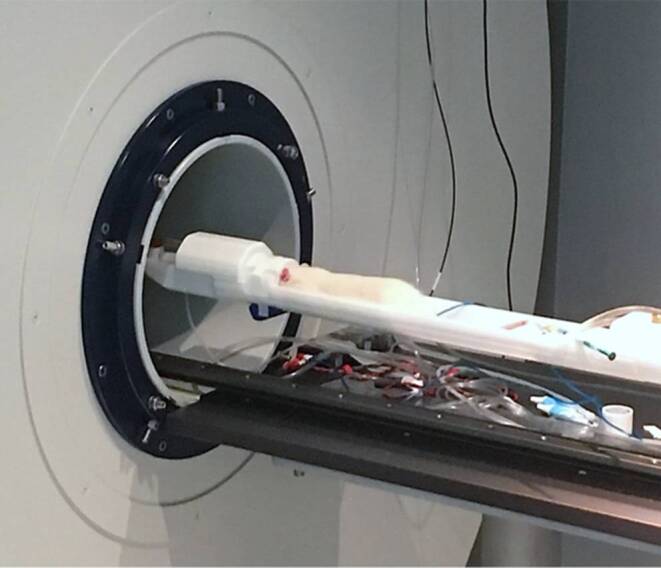
MRI (magnetic resonance imaging) in the rat using a 7 T MRI scanner dedicated for small animals MRT (Magnetresonanztomographie) einer Ratte mit einem 7‑Tesla-MRT-Scanner für Kleintiere

Quantitative assessment of local VEGFR‑2 expression with and without OTM was carried out by magnetic resonance imaging (MRI) in this study. MRI allows in vivo characterization and measurement of biological processes at the cellular level [28]. The examination by MRI was performed 4 days after insertion of the elastic band, since at this time the cellular tissue response during the induced OTM is reported to be most pronounced [27]. To assess the kinetics of a VEGF receptor 2 (VEGFR-2)-specific contrast agent within the periodontal ligament of the orthodontically moved upper rat molar compared to the contralateral control side, we obtained a corresponding validated molecular MRI marker (anti-VEGFR‑2 bound to albumin (Gd-diethylene penta-acetic acid [DTPA])—biotin, Rheal A. Towner, Advanced Magnetic Resonance Center, Oklahoma Medical Research Foundation, and The Oklahoma Center for Neurosciences, The University of Oklahoma Health Sciences Center, Oklahoma City, OK, USA). After 4 days of OTM, the rat was anesthetized with 4% isoflurane and maintained at 1.5% isoflurane. The head of the rat was fixed in a rat brain MRT surface coil and scanned on a preclinical 7 T MRI scanner (Fig. 1, ClinScan 70/30, Bruker, Ettlingen, Germany). Respiration was monitored by a pressure sensor and kept constant during the entire imaging procedure. Also the body temperature was kept constant employing a heating bed for the animal. A T1-weighted spin echo sequence (TR: 600 ms, TE: 10 ms, voxel size: 0.078 × 0.078 × 0.7 mm, acquisition time: 12:05 min) preceded the administration of the molecular marker. Dynamic contrast-enhanced MRI (DCE-MRI) was performed using a fast low angle shot (FLASH) sequence with the following parameters: TR: 2.92 ms, TE: 0.88 ms, flip angle: 25°, voxel size: 0.182 × 0.182 × 0.7 mm, acquisition time: 12 min 18 s and 100 measurements. Then, 200 μl of anti-VEGFR-2-BSA-Gd-DTPA-biotin was injected intravenously into a tail vein catheter (24G) after 30 s and over a time period of 10 s. After running the DCE sequence the above-mentioned T1-weighted MRI sequence was repeated and a T1-mapping sequence with the following parameters was scanned: acquisition time: 7:35 min, voxel size: 0.313 × 0.313 × 0.6 mm, TR: 40 ms, TE: 1.5 ms, flip angle 1: 5°, flip angle 2: 29° [9, 10, 25, 26].

### Analysis of MRI datasets and dynamic contrast-enhanced (DCE) MRI

The periodontal region of the first upper left and right molars within the T1-weighted images was analyzed. For determination of the volume enhancement after administration of anti-VEGFR-2-BSA-Gd-DTPA-biotin, T1-weighted images were segmented using a threshold of 100 for signal intensity. Relaxation times in the selected regions of interest (ROIs) were derived from T1-mapping and DCE-MRI resulted in the semiquantitative parameters area under the curve (AUC), time to peak (TTP) and washout (WO).

## Results

### Analysis of a T1-weighted mMRI of the upper jaw during orthodontic tooth movement

**Fig. 2:**
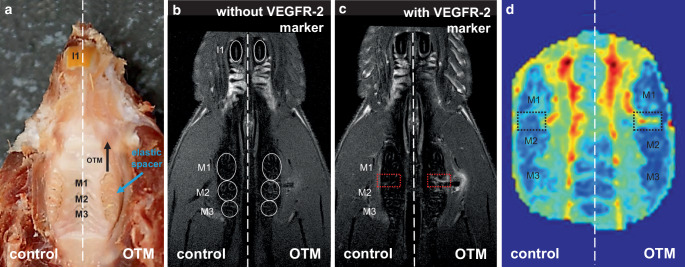
**a** Experimental anterior orthodontic tooth movement (OTM) of the first upper left molar (M1) by insertion of an elastic band (774-200-01, Dentaurum, Ispringen, Germany) between the first (M1) and second (M2) upper left molar according to the Waldo/Rothblatt method. **b** Axial T1-MRI section at the root level of the rat molars before i.v. injection of the VEGFR‑2 molecular marker. **c** Axial T1-MRI plane at the root level of the rat molars after i.v. injection of the VEGFR‑2 molecular marker. **d** Area under the curve (AUC) mapping of dynamic contrast-enhanced MRI (DCE-MRI). *VEGFR* vascular endothelial growth factor, *OTM* left jaw side with orthodontic tooth movement; *control* untreated right jaw side **a** Experimentelle kieferorthopädische Zahnbewegung (OTM) des ersten oberen linken Molaren (M1) durch Insertion eines elastischen Bandes (774-200-01, Dentaurum, Ispringen, Deutschland) zwischen dem ersten (M1) und zweiten (M2) oberen linken Molaren nach der Waldo/Rothblatt-Methode. **b** Axialer T1-MRT-Schnitt auf Wurzelniveau der Molaren der Ratte vor i.v.-Injektion des molekularen Markers VEGFR‑2. **c** Axiale T1-MRT-Ebene auf Wurzelniveau der Molaren der Ratte nach i.v.-Injektion des molekularen Markers VEGFR‑2. **d** AUC(Fläche unter der Kurve)-Kartierung der DCE-MRI. *VEGFR* „vascular endothelial growth factor“, *OTM* linke Kieferseite mit kieferorthopädischer Zahnbewegung; *Kontrolle* unbehandelte rechte Kieferseite

**Fig. 3:**
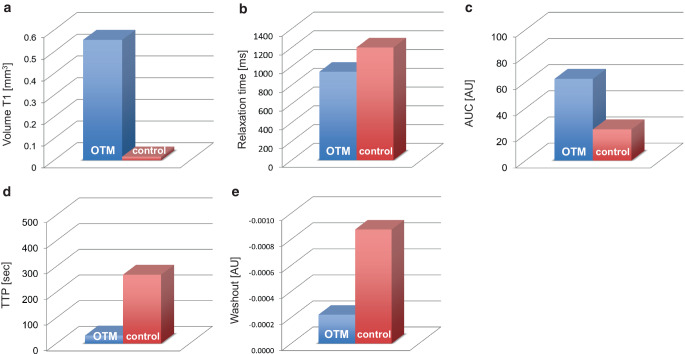
Results from the MRI analyses after i.v. injection of the VEGFR‑2 molecular marker at the left jaw side with orthodontic tooth movement (OTM) and the contralateral right untreated jaw side (control). **a** mMRI volume T1. **b** mMRI relaxation time. **c** Dynamic contrast-enhanced MRI (DCE-MRI) area under the curve (AUC). **d** Time to peak (TTP). **e** DCE-MRI washout rate. *AU* arbitrary units Ergebnisse der MRT-Analysen nach i.v.-Injektion des molekularen Markers VEGFR‑2 auf der linken Kieferseite mit kieferorthopädischer Zahnbewegung (OTM) und der kontralateralen rechten unbehandelten Kieferseite (Kontrolle). **a** mMRT-Volumen T1. **b** mMRI-Relaxationszeit. **c** DCE-MRT(dynamische kontrastmittelunterstützte MRT)-Bereich unter der Kurve (AUC). **d** Zeit bis zur Spitze (TTP). **e** DCE-MRT-Auswaschrate. *AU* arbiträre Einheiten

In this pilot study, we first assessed T1 volume, which represents the enrichment of the molecular marker for VEGFR‑2 in the indicated region of interest (ROI) (Fig. 2b,c). Intravenous injection of the molecular marker for VEGFR‑2 resulted in an increased T1 volume in the distal part of the periodontal ligament at the orthodontically moved upper first left molar compared to the contralateral control side (Fig. 2c and 3a). T1 relaxation time was reduced on the OTM side compared to the untreated side (Fig. 3b).

### Analysis of DCE-MRI datasets of the upper jaw during orthodontic tooth movement

Analysis of DCE-MRI datasets revealed an increased signal (area under the curve [AUC]) during OTM (Fig. 2d and 3c) compared to the contralateral control side, reflecting the degree of signal enhancement of the contrast agent in the tissue after molecular marker administration. Time to peak (TTP) was reduced at the OTM jaw side (Fig. 3d). In addition, we determined the washout rate and observed that the washout of the gadolinium-based contrast agent was accelerated during OTM (Fig. 3e).

## Discussion

The aim of this pilot study was to establish an imaging methodology that allows noninvasive assessment of VEGFR‑2 expression using MRI. This study was based on the idea that reduced local perfusion and concomitantly created hypoxic conditions during OTM result in increased VEGF-mediated angiogenesis. As current evidence supporting this assumption is scarce and actual perfusion and angiogenesis levels have not been evaluated before using advanced MRI imaging, we used a molecular MRI marker for VEGFR‑2 coupled to gadolinium biotin to help shed light on this question. Here, we describe an imaging protocol that clearly visualizes uptake of the VEGFR-2-specific contrast agent after OTM including quantitative measures for noninvasive determination of VEGFR‑2 expression.

In MRI analysis, T1-weighted images represent the standard sequences used to display variations in T1 relaxation times of various tissues in mice and humans. They are primarily dependent on the longitudinal relaxation of the net magnetization vector in a certain tissue [1]. In general, a radiofrequency pulse impacts on the alignment of spins, which are commonly orientated in a magnetic field, by transversing the orientation of the nuclear spins [1]. Accordingly, they return into the original alignment after a certain amount of time, which critically depends on the investigated tissue, as various tissues differ from each other in the duration needed to return to the initial orientation [1].

In our study, we observed an increased volume enhancement in T1-weighted imaging distally of the orthodontically moved first upper molar after injection of the VEGFR‑2 marker. In future studies, this may be used to confirm an increased local expression of this receptor hinting at increased VEGF-induced angiogenesis taking place at tension areas of the periodontal ligament, which has been assumed before [6, 15, 17, 18].

The time for realignment of nuclear spins in an external magnetic field after a high-frequency pulse is defined as T1-relaxation time [1]. Thus, the duration strongly depends on multiple variables like composition of the tissue, magnetic flux density, and strength of the magnetic field. A reduced relaxation time, which was observed in our pilot study during OTM distal to the orthodontically moved first upper molar after injection of the VEGFR‑2 marker, corresponds to an increased uptake of contrast agent.

In general, dynamic contrast-enhanced (DCE) MRI can determine the perfusion parameters of different tissues by calculation of T1-shortening evoked by a contrast agent, which diffuses through tissue (in this case the VEGFR‑2 molecular marker), by evaluating T1 changes in tissues over a certain amount of time. One main characteristic of contrast agents, like the most commonly used gadolinium, is a rapid dissemination into the plasma, where it circulates through tissues via blood flow, as they can very easily pass through the vascular endothelium due to their small size. In DCE analysis, the time course of contrast agent enrichment is quantified, which strongly depends on the vasculariszation of the investigated tissue.

Despite the statements that can be taken from the presented pilot experiment, there are several limitations that should also be mentioned at this point. First, we only investigated one animal in this study as proof-of-principle and to reduce animal suffering. Thus, generalizability of our findings cannot be shown at the statistical level. Furthermore, no MRI control prior to insertion of the elastic bands could be recorded as a nonforce control to contrast our findings to. Given the fact that the procedure of MRI scanning is very time-consuming and the corresponding long-term anesthesia applied, MRI scanning prior to insertion may have affected the results, as the final MRI scan would have taken place only 4 days later due to the limited time of orthodontic tooth movement. Finally, we used an elastic band instead of a nickel–titanium (NiTi) coil to induce experimental tooth movement, as the appliance had to be in place during MRI measurements and NiTi coil springs would have created MRI artifacts. Therefore, we could not control the applied force during OTM. Additional examinations such as histological staining will be essential in the future to corroborate VEGFR‑2 expression during orthodontic tooth movement in rats.

Nevertheless, we observed an increased AUC at the region of interest distally of the orthodontically moved first upper molar, whereas the time to peak and washout rate were all decreased compared to the untreated contralateral jaw side in our pilot study. These preliminary findings indicate that perfusion of this area was increased during OTM. According to current theory, blood perfusion is believed to be predominantly reduced at compression areas of the periodontal ligament with the tooth roots pressing against the alveolar bone surfaces of the alveolar sockets thus compressing the periodontal ligament (PDL) in-between [12, 13, 15]. A possible explanation for the observed increased blood perfusion in tension areas of the PDL could be the fact that VEGF-mediated angiogenesis has taken place within the OTM phase of four days, thus increasing the number of periodontal blood vessels, which has been postulated before [6, 12, 13, 15, 17].

## Conclusions

Our results from this pilot study, which for the first time used a VEGFR‑2 (vascular endothelial growth factor receptor 2)-specific MRI (magnetic resonance imaging) contrast agent to assess tissue effects during OTM (orthodontic tooth movement), indicate that noninvasive MRI assessment of VEGFR‑2 expression within the jaw and PDL (periodontal ligament) after OTM is feasible. Our preliminary results suggest that local perfusion is actually increased in tension areas of the PDL during OTM. This is most likely due to increased VEGF-mediated angiogenesis within the periodontal ligament at tension areas. Taken together, our pilot study provides a novel method and quantitative imaging markers to noninvasively assess VEGFR‑2 expression during OTM.
